# Association and predictive value analysis for metabolic syndrome on systolic and diastolic heart failure in high-risk patients

**DOI:** 10.1186/1471-2261-14-124

**Published:** 2014-09-24

**Authors:** Zi-Hui Tang, Lin Wang, Fangfang Zeng, Keqin Zhang

**Affiliations:** Department of Endocrinology and Metabolism, Shanghai Tongji Hospital, Tongji University School of Medicine, Building 2nd, NO.389 Xincun Road, Shanghai, 200063 China; Department of Cardiology, Fudan University Huashan Hospital, Shanghai, China

**Keywords:** Metabolic syndrome, Diastolic heart failure, Systolic heart failure, High-risk patients, association

## Abstract

**Background:**

The purpose of this study was, in high-risk patients, to simultaneously estimate the effect of metabolic syndrome (MetS) on diastolic or systolic heart failure (DHF or SHF), to evaluate MetS predictive value for both outcomes.

**Method:**

We retrospective enrolled 347 high-risk patients who were scheduled to undergo coronary angiography. They were categorized into DHF cases, SHF cases and reference group. The association of MetS with DHF or SHF was assessed by multinomial logistic regression model. The shared contributor to both outcomes was estimated by bivariate association analysis. The predictive performance of MetS severity score was evaluated using the area under the receiver-operating characteristic curve (AUC).

**Result:**

Hypertension (HT) and triglycerides (TG) were detected to independently associate with DHF (*P* = 0.044 and 0.049, respectively), while HT and fasting plasma glucose (FPG) independently associate with SHF (*P* = 0.036 and 0.016, respectively). Bivariate association analysis showed that HT as a shared predictor to both outcomes (*P* = 0.028). MetS severity score significantly associated with DHF or SHF independently (*P* = 0.004 and 0.043, respectively), and was a shared predictor to both outcomes (*P* = 0.049), and showed a high value in predicting DHF and SHF (AUC = 0.701 and 0.722, respectively).

**Conclusion:**

Our findings signify that MetS is an independently shared predictor of DHF and SHF, and HT is also independently associated with both outcomes in high-risk patients. Prevalence of DHF or SHF trends to increase with increasing MetS severity showing high predictive value for both outcomes.

## Background

Heart failure (HF) occurs when the heart is unable to provide sufficient pump action to distribute blood flow to meet the needs of the body [1]. Systolic heart failure refers to failure of the pump function of the heart, which characterized by a decreased ejection fraction (less than 45%) [2, 3]. Diastolic heart failure is generally described as the failure of the ventricle to adequately relax and typically denotes a stiffer ventricular wall. DHF and SHF can be attributed to multiple factors that are mainly linked to metabolic disturbances [4]. Metabolic syndrome (MetS) refers to a constellation of cardiovascular disease risk factors including obesity and abdominal fat distribution, disorders of glucose and lipid metabolism, and hypertension [5]. MetS components strongly associated with DHF and SHF, which leads to stiffening of LV resulting to diastolic or systolic dysfunction [4, 6]. Additionally, hypertension (HT), diabetes mellitus (DM), and obesity were found to adversely affect cardiac structure and function [6–9].

Patients with established cardiovascular disease or additional high-risk cardiovascular disease characteristically have HT, DM and hyperlipidemia. MetS, DHF and SHF trend to be co-prevalent in high-risk patients who accounted for more than a half of the hospitalization patients in department of cardiovascular disease [10]. High-risk patients with diastolic and systolic HF were found to have high morbidity and mortality. It is important to clarify the relationship of MetS, DHF and SHF in high risk patients because this information can be of benefit to clinicians in the prediction, prevention and treatment and of DHF and SHF. In addition, previous studies were conducted to explore the relationship of MetS and DHF or SHF in respective reference sample, but not a shared reference sample [8, 9]. The multinomial logistic regression (LR) includes several LR models to estimate the associations between predictors and each of outcomes as compared with reference category simultaneous [11]. So regression coefficients may differ per outcome.

However, in high-risk patients, the extent to which clustering components of the MetS predicting DHF and SHF in an entire sample has not been well characterized. Little document has been found to reported shared predictors to both outcomes. In addition, the predictive value of MetS severity for DHF and SHF can be developed in prospective cohort study or the cross-sectional study. The purpose of this study was, in high-risk patients, to estimate effects of MetS or its components with DHF and/or SHF simultaneous in an entire sample, to evaluate shared predictors to both DHF and SHF, and assess the predictive value of MetS severity for DHF and SHF.

## Methods

### Study population

We retrospectively recruited 347 consecutive symptomatic Chinese patients with suspected myocardial ischemia scheduled for coronary angiography between February 2009 and May 2011 at the Huashan Hospital of Fudan University, China. Patients were excluded from the study to eliminate potential confounding factors which may influence systolic and diastolic heart function. Exclusion criteria included the following: 1) history or findings of significant valvular heart disease (i.e. more than a mild valvular insufficiency or stenosis), hyperthyroidism or hypothyroidism and dilated or hypertrophic cardiomyopathy; 2) atrial fibrillation; 3) pregnancy or lactation; and/or 4) a major systemic illness such as systemic lupus erythematosus. Written consent form was obtained from all patients before the study. The present study was approved by the Ethics Committee of the Huashan Hospital, Shanghai, China. This study was a cross-sectional study performed in inpatients.

MetS was diagnosed according to the definition of MetS recommended by the International Diabetes Federation (IDF, 2005) consensus worldwide definition of the metabolic syndrome is [12]: Central obesity (defined as waist circumference with ethnicity-specific values: ≥ 90 cm for male and ≥ 80 cm for female) AND any two of the following: (1) Raised triglycerides: > 1.70 mmol/L, or specific treatment for this lipid abnormality; (2) Reduced HDL cholesterol: < 1.03 mmol/L in males, < 1.29 mmol/L in females, or specific treatment for this lipid abnormality; (3) Raised blood pressure (BP): systolic BP > 130 or diastolic BP >85 mm Hg, or treatment of previously diagnosed hypertension; (4) Raised fasting plasma glucose (FPG) > 5.60 mmol/L, or previously diagnosed type 2 diabetes. In addition, if BMI is > 30.0 kg/m^2^, central obesity can be assumed and waist circumference does not need to be measured. MetS severity was scored on a scale of 0 to 4 according to the number of MetS components. Seven subjects met all 5 MetS criteria, and their MetS score was set to 4. The subjects were interviewed for the documentation of medical histories and medications, history of smoking habits, laboratory assessment of cardiovascular disease risk factors and standardized echocardiographic examination. Body mass index (BMI) was calculated as the weight in kilograms divided by the square of height in metres. SBP and DBP values were the means of two physician-obtained measurements on the left arm of the seated participant. Hypertension (HT) was diagnosed if the BP ≥ 140/90 mmHg and/or the patient was undergoing current antihypertensive therapy. Diabetes (DM) was defined by oral glucose tolerance test (OGTT) and either glycosylated hemoglobin (HbAlc) ≥ 6.5% or the use of insulin or hypoglycaemic medications. Cardiovascular artery disease (CAD) was defined as [13]: (1) history and/or treatment for angina and/or myocardial infarction; and/ or (2) history of coronary artery revascularization procedures and/or coronary angiography with ≥50% stenosis in one or more of the major coronary arteries.

### Laboratory assays

Fasting plasma glucose (FPG) was quantified by the glucose oxidase procedure; HbA1c was measured using ion-exchange high-performance liquid chromatography (HPLC; Bio-Rad, Hercules, CA, USA). Serum total cholesterol (TC), high-density lipoprotein (HDL) cholesterol, triglyceride (TG) levels, creatinine (Cr), and uric acid (UA) level were measured using an enzymatic method with a chemical analyzer (Hitachi 7600–020, Tokyo, Japan). Low-density lipoprotein (LDL) cholesterol levels were calculated using the Friedewald formula, and the creatinine clearance rate (Ccr) was calculated using the Cockcroft-Gault formula. The day-to-day and inter-assay coefficients of variation at the central laboratory in our hospital for all analyses were between 1% and 3%.

### Echocardiographic measurement

Echocardiography examinations were performed with a Vingmed System 5 Doppler echocardiographic unit (GE Vingmed Ultrasound, Horten, Norway). Conventional echocardiography measurements were performed according to American Society of Echocardiography guidelines. LV mass (LVM) was calculated using the Devereux formula. LVM was corrected for body surface area (BSA) to obtain the LVM index (LVMI). Left atrial diameter (LAD) and aortic root dimension (AOD) were also measured. LV systolic function was assessed using LV ejection fraction (LVEF). Diastolic function was assessed by determining the E-to-A ratio (E/A) and deceleration time (DT), where E and A represent the early and late ventricular filling velocities respectively. The diagnosis of SHF requires the following criteria: (1) Presence of signs and/or symptoms of chronic HF; (2) Presence of abnormal LV systolic function (LVEF < 45%). The definition of DHF was recommended by the European Society Cardiology guidelines in 2008 [3]. The diagnosis of DHF requires three conditions to be satisfied: (1) presence of signs and/or symptoms of chronic HF; (2) presence of normal or only mildly abnormal LV systolic function (LVEF ≥50%); (3) evidence of diastolic dysfunction (abnormal LV relaxation or diastolic stiffness). Diastolic function of LV was evaluated on the basis of the ventricular filling pattern to detecting abnormalities of diastolic function or filling in patients with HF. Normal LV diastolic function was defined as E/A ratio >1 and 160 ms < DT <240 ms. LV diastolic dysfunction was defined as the following criteria: (1) E/A ratio < 1 and DT ≥ 260 ms or (2) E/A ratio > 2 and DT < 150 ms.

### Data analysis

Data were checked for normality and described as mean ± SD or median unless stated otherwise. Kolmogorov-Smirnov Test was used to determine whether continuous variables followed a normal distribution. Variables that were not normally distributed were log-transformed to approximate normal distribution for analysis. The characteristics of subjects according to SHF, DHF and control were assessed using the one-way analysis of variance (ANOVA) for continuous variables and the *χ*^2^ test for categorical variables. Univariate LR was performed to determine the variables associated with outcomes and to estimate confounding factors possibly disturbing the relationship between MetS and SHF or DHF. Univariate association between candidate predictors and the different outcome categories were estimated using multinomial LR analysis which allows for simultaneous estimation of the probability of SHF and DHF compared with control as reference category. The multinomial LR analysis includes several LR models simultaneous to estimate the associations between predictors and each of outcomes compared with reference category simultaneous so that regression coefficients may differ per outcome [11]. Multivariable LR controlling for confounders was carried out to determine contribution of independent variables to SHF or DHF. Potential confounders including age, gender, smoking, HR, UA, Ccr, LVMI and CAD were controlled in the regression model. The models were analyzed after substituting the continuous variables related to MetS components with their dichotomous counterparts in the models. Variables were entered into the backward stepwise regression models if a P value < 0.10 was obtained. Bivariate association analysis based on generalized linear model may treat correlations of outcomes so as to be more power to detect the shared contributors to outcomes as compared to univariate association analysis [14]. Generalized linear model was performed to include dependences of both SHF and DHF simultaneous to identify shared predictors. The predictive performance of MetS severity score was evaluated with respect to the area under the curve (AUC) in a receiver operating characteristics (ROC) curve. Odds ratios (OR) with 95% confidence intervals (CI) were calculated for the relative risk of MetS with SHD or DHF. Results were analyzed using the Statistical Package for Social Sciences for Windows version 16.0 (SPSS, Chicago, IL, USA). Tests were two-sided and a *P*-value of < 0.05 was considered significant.

## Results

Baseline clinical characteristics of the 347 subjects were grouped according to SHF, DHF and control (Table 1). The total sample included 208 males and 139 females (mean age, 57.18 ± 12.94 years) in total sample. Gender, height, PBG, HbAlc, LDL and TC levels were similar among the three groups (p > 0.05), while the other demographic parameters and biochemistry variables were significantly different (p < 0.05). LVEF were significantly different among the three groups. The prevalence of HT, DM, MetS and CAD were 71.18%, 49.26%, 26.51% and 24.78% in the patients, respectively. The four chronic diseases were more prevalent in SHF group than the other two groups (p < 0.05). In the high-risk patients, the CAD prevalence was no significant difference among three groups (P = 0.208). The use of oral medications were significant different among the three groups (p < 0.05 for all). The number of patients with DHF and SHF were 97 and 126 accounted for 27.95% and 36.71% in the patients, respectively. SHF was more prevalent than DHF in total subjects. The subjects in New York Heart Association (NYHA) class IV and III was 11.11% and 12.98% in SHF groups, respectively. The subjects in NYHA class III was 12.37% in DHF groups.

**Table 1:** **Characteristics of subjects**

Variable	Total (n = 347)	Reference (n = 124)	DHF (n = 97)	SHF (n = 126)	***P***value
Age	57.18 ± 12.94	48.36 ± 12.34	60.28 ± 10.87	63.48 ± 9.93	<0.001
Gender (Female, %)	139 (40.06%)	52 (41.94%)	39 (40.21%)	48 (38.1%)	0.662
BMI	23.80 ± 3.88	22.93 ± 3.28	24.22 ± 3.44	24.35 ± 4.57	0.007
WC	82.64 ± 7.82	78.97 ± 6.21	82.91 ± 7.18	86.04 ± 7.09	<0.001
SBP	127.42 ± 19.01	120.12 ± 14.57	134.46 ± 20.75	129.24 ± 19.17	<0.001
DBP	77.59 ± 11.47	75.07 ± 10.17	80.63 ± 12.84	77.73 ± 11.07	0.002
HR	74.77 ± 14.51	70.84 ± 10.75	74.4 ± 13.91	79.65 ± 17.24	<0.001
Laboratory assay					
FPG	6.23 ± 2.46	5.80 ± 2.63	6.38 ± 2.71	6.54 ± 1.99	0.047
PBG	8.92 ± 4.35	8.27 ± 4.54	9.36 ± 4.28	9.04 ± 4.28	0.402
HbAlc	6.81 ± 1.99	6.73 ± 2.55	6.96 ± 2.13	6.75 ± 1.44	0.805
TC	4.46 ± 1.08	4.44 ± 1.16	4.59 ± 1.08	4.39 ± 0.98	0.373
TG	1.70 ± 1.37	1.53 ± 1.20	1.87 ± 1.35	1.74 ± 1.54	0.176
HDL	1.09 ± 0.29	1.17 ± 0.31	1.09 ± 0.28	1.00 ± 0.26	<0.001
LDL	2.58 ± 0.87	2.5 ± 0.91	2.64 ± 0.89	2.62 ± 0.8	0.445
SCr	80.31 ± 39.9	68.74 ± 14.72	74.71 ± 16.48	100.4 ± 42.1	<0.001
Ccr	86.56 ± 31.82	101.08 ± 26.41	86.86 ± 25.59	71.31 ± 34.35	<0.001
UA	0.36 ± 0.12	0.32 ± 0.08	0.36 ± 0.12	0.4 ± 0.13	<0.001
Echocardiography					
LAD	38.13 ± 6.24	33.85 ± 3.4	37.44 ± 5.05	42.91 ± 5.95	<0.001
LVMI	126.83 ± 48.78	98.47 ± 25.21	118.08 ± 40.75	161.89 ± 51.01	<0.001
EF	55.53 ± 14.50	65.55 ± 5.22	65.68 ± 6.39	38.00 ± 5.77	<0.001
DT	203.92 ± 54.74	189.19 ± 23.19	231.82 ± 69.73	163.86 ± 50.97	<0.001
Medical history					
HT (yes, %)	247 (71.18%)	57 (45.97%)	73 (75.26%)	117 (92.86%)	<0.001
DM (yes, %)	166 (49.26%)	36 (29.03%)	53 (54.64%)	77 (61.11%)	<0.001
MetS (yes, %)	92 (26.51%)	16 (12.9%)	30 (30.93%)	46 (36.51%)	<0.001
CAD (yes, %)	86 (24.78%)	24 (19.35%)	26 (26.8%)	36 (28.57%)	0.208
Smoking (yes, %)	123 (38.2%)	40 (32.26%)	27 (27.84%)	56 (44.44%)	<0.001
Medical therapy					
Anti-HT	232 (66.86%)	52 (41.94%)	39 (40.21%)	48 (38.1%)	<0.001
Anti-DM	119 (35.74%)	27 (21.77%)	43 (44.33%)	49 (38.89%)	<0.001
Anti-HF	89 (25.65%)	1 (0.81%)	10 (10.31%)	78 (61.9%)	<0.001
Anti-Lip	113 (33.73%)	28 (22.58%)	23 (23.71%)	62 (49.21%)	<0.001

Note: BMI- Body mass index, WC- waist circumference, SBP- systolic blood pressure, DBP- diastolic blood pressure, MetS- metabolic syndrome, HT- Hypertension, DM- Diabetes, FPG- fasting plasma glucose, PBG- plasma blood glucose, HbA1c- glycated hemoglobin, TC- serum total cholesterol, HDL- high-density lipoprotein cholesterol, TG- triglyceride, UA- uric acid, LDL- low density lipoprotein cholesterol, Ccr- creatinine clearance rate, Cr- creatinine, LVMI- left ventricular mass index, LAD- left atrial diameter, DT- deceleration time, LVEF- left ventricular ejection fraction, HF- heart failure.

### Components of MetS v.s SHF and DHF

Univariate association analysis using multinomial LR to include single independent variable showed that MetS and its components significantly associated with SHF or DHF (P < 0.05 for all, data not shown). Backward stepwise multinomial LR model to include components of MetS controlling for covariates of age, gender, smoking, HR, Ccr, UA, LVMI and CAD, indicated that three components of MetS – FPG, HT and TG significantly associated with SHF or DHF independently (Table 2). HT and TG were detected to independently associate with DHF (*P* = 0.044, OR = 1.34, 95% CI 0.61-2.93 for HT and *P* = 0.049, OR = 2.07, 95% CI 1.20-3.59 for TG), while HT and FPG independently associate with SHF (*P* = 0.036, OR = 3.59, 95% CI 1.08-11.88 for HT and *P* = 0.016, OR = 3.75, 95% CI 1.28-10.95 for FPG). LR models demonstrated that HT associated with DHF or SHF. Its regression coefficient was significant greater in LR model for SHF as compared with that for DHF (P = 0.022, data not shown). In patients with HT, the OR of DHF was 1.34, while OR of SHF was 3.59. To evaluate shared predictor of both SHF and DHF, bivariate association analysis, with both outcomes as dependent variable simultaneous, based on generalized linear model to include all components of MetS controlling for confounders was conducted to confirm HT to be a shared predictor of both outcomes (Wilks' λ = 0.970, *P* = 0.028 Table 3).

**Table 2:** **Final model using backward stepwise multinomial logistic regression analysis to include MetS components for SHF and DHF**

Model	Variables	***β***	SE	***P***value	***OR***	95% CI
DHF v.s Control	Age	0.655	0.165	<0.001	1.92	1.39-2.66
	LVMI	0.015	0.006	0.013	1.01	1.00-1.02
	HR	0.469	0.41	0.252	1.60	0.71-3.56
	FPG	0.049	0.481	0.919	1.05	0.40-2.69
	HT	0.292	0.109	0.044	1.34	1.08-1.66
	TG	0.728	0.280	0.049	2.07	1.20-3.59
	Intercept	-6.346	1.132	<0.001		
SHF v.s Control	Age	0.718	0.211	0.001	2.05	1.35-3.09
	LVMI	0.037	0.007	<0.001	1.03	1.02-1.05
	HR	1.565	0.439	<0.001	4.78	2.02-11.31
	FPG	1.322	0.547	0.016	3.75	1.28-10.95
	HT	1.278	0.611	0.036	3.59	1.08-11.88
	TG	-0.02	0.471	0.967	0.98	0.39-2.466
	Intercept	-12.024	1.719	<0.001		

Note: Age, gender, smoking, HR, Ccr, UA, LVMI, CAD and medical therapy are stated to enter multinomial logistic regression analysis. HT- Hypertension, FPG- fasting plasma glucose, TG- triglyceride, LVMI- left ventricular mass index, HR- heart rate.

**Table 3:** **Bivariate association analysis to detect shared predicator for SHF and DHF**

Model	Variable	Wilks' λ	***F***statistic	***P***value
Model 1	BMI	0.995	0.592	0.554
	HT	0.970	3.631	0.028
	FPG	0.977	2.894	0.057
	TG	0.972	3.525	0.031
	HDL	0.991	1.102	0.335
Model 2	MetS	0.934	1.971	0.049

Note: Model 1 including all MetS components adjusted for age, gender, smoking, HR, Ccr, UA, LVMI, CAD and medical therapy; Model 2 including MetS adjusted for age, gender, smoking, HR, Ccr, UA, LVMI, CAD and medical therapy.

### MetS severity v.s SHF and DHF

**Figure 1:**
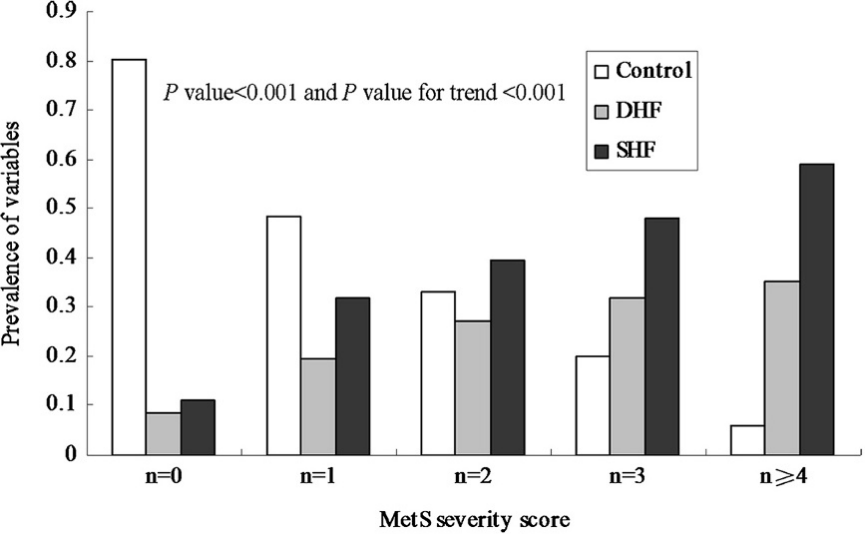
**The prevalence of diastolic heart failure (DHF) and systolic heart failure (SHF) in groups according to metabolic syndrome (MetS) severity score.** White bar represent proportion of control, grey bar represent prevalence of DHF and black bar represent prevalence of SHF.

**Figure 2:**
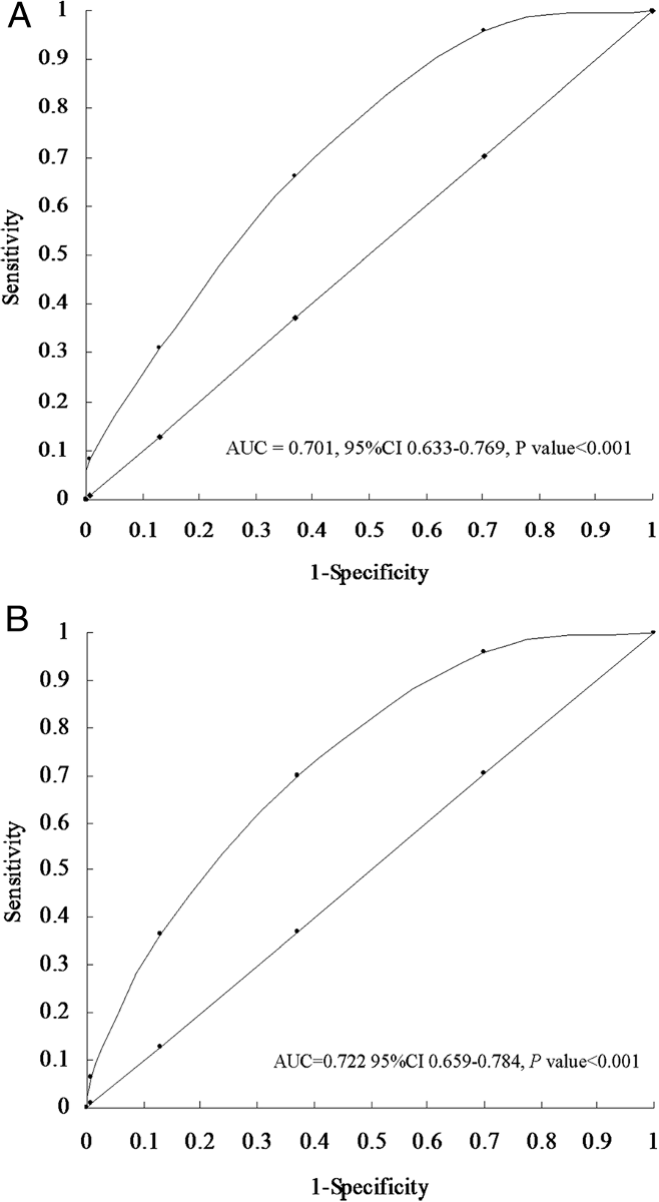
**Performance of MetS severity score in predicting DHF and SHF. A**: Performance of MetS severity score in predicting DHF, AUC of ROC analysis was 0.701, 95% CI 0.633-0.759 P < 0.001; **B**: Performance of MetS severity score in predicting SHF, AUC of ROC analysis was 0.722, 95% CI 0.659-0.784 P < 0.001.

The prevalence of DHF and SHF was increased with the increasing MetS severity score respectively (Figure 1). The prevalence of DHF was 8.69%, 19.41%, 27.36%, 32.00% and 35.29% in five groups according to MetS severity score, respectively. Similarly, the prevalence of SHF also increased with increasing MetS severity score (*P* value for trend < 0.001). Patients with SHF accounted for 58.82% in group with the top MetS severity score. Figure 1 showed that as MetS severity scores increased, prevalence of SHF and DHF also increased (*P* for trend < 0.01). In addition, SHF prevalence was higher in each group than that of DHF. To estimate the association of MetS severity with SHF or DHF, univariate association analysis to include single predictor indicated MetS severity score significant association with SHF or DHF (P < 0.05 for all, data not shown). Backward stepwise multinomial LR model also signified that MetS severity score significantly associated with DHF or SHF independently (*P* value = 0.004, OR = 1.64, 95% CI 1.16-2.31 for DHF and *P* value = 0.043, OR = 1.13, 95% CI 0.89-1.98 for SHF Table 4). In patients with MetS severity score of 1, the OR of DHF was 1.64, and OR of SHF was 1.13. Bivariate association analysis demonstrated that MetS severity score was a shared contributor to both DHF and SHF (Wilks' λ = 0.934, *P* value = 0.049 Table 3). To evaluate the predictive performance of MetS severity score for DHF and SHF, the area under the curve (AUC) in a receiver operating characteristics (ROC) curve has been calculated. The AUC was 0.701 (95% CI, 0.633-0.767, *P* value <0.001, Figure 2A) and 0.722 (95% CI, 0.659-0.784, *P* value <0.001, Figure 2B) for DHF and SHF, respectively, indicating MetS severity score has a high value in predicting DHF and SHF.

**Table 4:** **Final model using backward stepwise multinomial logistic regression analysis to include MetS for SHF and DHF**

Model	Variables	***β***	SE	***P***value	***OR***	95% CI
DHF v.s Control	Age	0.672	0.156	<0.001	1.96	1.44-2.65
	LVMI	0.015	0.006	0.008	1.02	1.00-1.02
	HR	0.914	0.381	0.017	2.49	1.18-5.26
	Anti-lipids drug	-0.706	0.412	0.087	0.49	0.22-1.10
	MetS	0.497	0.175	0.004	1.64	1.16-2.31
	Intercept	-5.317	0.888	<0.001		
SHF v.s Control	Age	0.690	0.195	<0.001	1.99	1.36-2.92
	LVMI	0.040	0.007	<0.001	1.04	1.02-1.05
	HR	1.807	0.414	<0.001	6.09	2.70-13.72
	Anti-lipids drug	0.328	0.450	0.466	1.39	0.57-3.35
	MetS	0.286	0.105	0.043	1.33	1.08-1.64
	Intercept	-9.998	1.182	<0.001		

Note: Age, gender, smoking, HR, Ccr, UA, LVMI, anti-Lips, anti-hypertension and lowering lipids drugs and CAD are stated to enter multinomial logistic regression analysis. MetS- metabolic syndrome, LVMI- left ventricular mass index, HR- heart rate.

## Discussion

We carried out a cross-sectional study to evaluate the effect of metabolic factors on both DHF and SHF in Chinese high-risk patients. Of a total of 347 subjects, 71.18%, 49.2% and 24.78% patients had HT, DM and CAD, respectively. Patients with DHF and/or SHF were present in 64.27% of total sample. The CAD prevalence was no significant among three groups. This is partly because we recruited high-risk patients who were with established CAD or additional high-risk cardiovascular disease. Most of the demographic factors, biochemical characteristics and echocardiographic measurements were significantly differed among the three groups. In the present study, Doppler echocardiography has become a well accepted, reliable noninvasive tool to measure LV diastolic function in order to diagnose DHF.

The main finding of this study was that MetS strongly and independently associated with DHF and SHF, as an independent shared predictor with a high value in predicting both outcomes in high-risk patients. Backward stepwise multinomial LR analysis implied that MetS was independently associated with both DHF and SHF, respectively. The approach includes two LR models to simultaneous estimate regression coefficients in the same sample, which can indicate difference in associations between MetS and the two outcomes. In patients with MetS severity score of 1, OR for DHF was 1.64, while 1.33 was for SHF (Table 4), which suggested that patients with MetS were greater at risk for DHF than patient with SHF. Moreover, bivariate association analysis based on generalized linear model is applied for identifying shared predictors to multi-outcomes, which can analysis correlations of outcomes and more efficiently and steadily integrate information of outcomes. The results from the approach showed strong evidence to support the hypothesis that MetS was a shared predictor to both outcomes. Specially, the prevalence of DHF and SHF increased with increasing MetS severity score, respectively. HT, insulin resistance or obesity were associated with LV diastolic dysfunction or DHF in different populations [15]. In addition, MetS was independently correlated with DHF or SHF in different subgroups such diabetic, non-diabetic or hypertension patients [16]. The clustering of cardiovascular risk factors in MetS indicated that multiple complex metabolic reactions involved in glycotoxicity, lipotoxicity, altered insulin signaling, increased cytokine activity and interstitial deposition of triacylglycerol, which may all directly or indirectly to impact on myocardial function [17, 18]. Moreover, these metabolic risk factors lead to reduced energy availability, and have an additive and adverse effect on endothelial dysfunction [19]. In the present study, AUC was calculated to show that MetS severity score has a high value in predicting DHF or SHF. When patients with MetS severity score of up to 4, the prevalence of heart failure consisted of DHF and SHF was near 90% in high-risk patients. This finding indicates that the severity of MetS is linked to the progression of DHF and SHF. However, in the present study, we scored the MetS severity by simply using the number of MetS criteria. We did not consider the weights of MetS componenAnother interesting finding was that HT and TG were found to contribute to DHF, while HT and FPG contribute to SHF. Results from bivariate association analysis supported that HT was a shared contributor to both outcomes. This finding is inconsistent with those of some earlier studies, which had revealed that BMI and lipid profiles were significantly associated with systolic and diastolic parameters and the structure and functions of the LV [17]. In the present study, BMI and HDL were not significantly associated with DHF or SHF. This is partly because contributions of separated MetS component could not be detected in the present study, which had a moderate sample size. Another possible cause is that the present study population was differed from previous studies. In addition, greater regression coefficient in LR model for SHF was found to support this hypothesis that HT may be more impact on the progression of SHF than that of DHF. The observations will provide evidence for clinicians to better understand and treat patients in this specific subgroup. FPG was also found to independently associate with SHF in backward stepwise multinomial LR model. Previous studies reported that FPG was an importance independent predictor of LV systolic dysfunction [20, 21]. In the present study, TG has been reported to associate with DHF but not with SHF. Previous studies have also reported similar results [22]. Bivariate association analysis denoted that TG was simultaneous association with both outcomes (Table 3). No consistent results have been found in backward stepwise multinomial LR analyses. This is partly because more high-risk patients with SHF were regularly treated with anti-lipids drugs as compared with patients with DHF (6.87% v.s 18.51%, P value < 0.001), this may have influenced the true value of TG resulting to make it difficult to detect effect of TG on DHF although multivariate regression model controlling for the confounding factor. The exact mechanism underlying the association between TG and DHF has not been fully elucidated. In the present study, we did not propose to delineate the mechanisms via TG modifies metabolic factors on development of DHF.

Several limitations of the study deserve comment. First, the design of the present study was based on hospital-based cross-sectional study, which is susceptible to selection bias. Second, the sample is not representative should also be stressed, and the sample size was moderate, limiting its ability to detect more significant association results. Third, the multiple regression models indicated only a moderate influence of MetS on DHF and SHF. Other environmental and genetics factors may contribute to the unexplained variation in DHF and SHF prevalence. Forth, the association between insulin resistance and the two outcomes was not analyzed in the present study. This is because data on fasting blood insulin were seriously missing. Similar data interpretation was performed in blood BMP levels. Finally, it is important to mention that our study concerned Chinese individuals and our findings may not be relevant to those of other ethnic.

## Conclusion

Our findings signify that MetS is an independently shared predictor of DHF and SHF, and HT is also an independently shared predictor to both outcomes, and TG and FPG is independently association with DHF and SHF, respectively. In addition, MetS has a high value in predicting DHF and SHF. There is a tendency toward increased prevalence of DHF and SHF with increasing MetS severity score. This supports the hypothesis that MetS areis involved in the regulation of progression of DHF and SHF. The present observation provides novel insight into future biological function researches.

## Abbreviations

ACEI: Angiotensin-converting enzyme inhibitor
Alb/Cr: Albumin/urinary creatinine ratio
AOD: Aortic root dimension
BMI: Body mass index
BSA: Body surface area
CAD: Cardiovascular artery disease
Ccr: Creatinine clearance rate
CI: Confidence intervals
Cr: Creatinine
DBP: Diastolic blood pressure
DHF: Diastolic heart failure
DM: Diabetes
DT: Deceleration time
E/A: E-to-A ratio
FPG: Fasting plasma glucose
GLM: General linear model
HbAlc: Glycosylated hemoglobin
HDL: High-density lipoprotein cholesterol
HF: Heart failure
HOMA-IR: Homeostasis model assessment insulin resistance estimate
HT: Hypertension
IDF: International Diabetes Federation
LDL: Low-density lipoprotein cholesterol
LV: Left ventricle
LVEF: Left ventricular ejection fraction
LVM: Left ventricular mass
LVMI: Left ventricular mass index
MetS: Metabolic syndrome
MLR: Multivariable logistic linear regression
OGTT: Oral glucose tolerance test
OR: Odds ratios
PBG: Postprandial blood glucose
SHF: Systolic heart failure
TC: Serum total cholesterol
TG: Triglyceride
WC: Waist circumference
UA: Uric acid.
